# The prognostic value of tumor markers doubling times in medullary thyroid carcinoma - preliminary report

**DOI:** 10.1186/1756-6614-3-10

**Published:** 2010-11-03

**Authors:** Tomasz Gawlik, Andrea d'Amico, Sylwia Szpak-Ulczok, Aleksander Skoczylas, Elżbieta Gubała, Anna Chorąży, Kamil Gorczewski, Jan Włoch, Barbara Jarząb

**Affiliations:** 1 Department of Nuclear Medicine and Endocrine Oncology, Maria Sklodowska-Curie Memorial Cancer Center and Institute of Oncology, Gliwice Branch, Poland; 2 Oncology and Reconstructing Surgery Clinic, Maria Sklodowska-Curie Memorial Cancer Center and Institute of Oncology, Gliwice Branch, Poland

## Abstract

**Introduction:**

Calcitonin (Ct) and carcinoembrional antigen (CEA) are widely used as tumor markers for the post-operative follow-up of patients with medullary thyroid carcinoma (MTC).

In patients with elevated serum Ct and CEA their dynamics can be described by calculating the doubling time (DT) - the time, they need to double the serum concentration. Previous reports concluded that the Ct and CEA DT have prognostic value in MTC patients.

**Patients and methods:**

We retrospectively analyzed data of 70 MTC patients with elevated serum Ct or CEA. In total, doubling times were calculated and the DT of the less favorable marker was used to stratify the patients into the low- and high-risk group with the cut-off value of 2 years. The survival analysis was performed using Cox proportional hazard method.

**Results:**

The doubling time < = 2 years of the less-favorable marker had significant prognostic impact for recurrence-free survival, HR = 2.61 (1.43-4.71) and overall survival, HR = 8.99 (3.51-23.04).

**Conclusions:**

The calcitonin and carcinembrional antigen doubling times of less than two years are negative prognostic factors for MTC recurrence-free and total survival in patients with persistent or recurrent disease. They may be used as predictive factors for more intensive search of disease localization in asymptomatic hypercalcitoninemia and for therapy choice in symptomatic disease.

## Introduction

Medullary thyroid carcinoma (MTC) originates from thyroid C-cells. The most important way of it's treatment is total surgical excision of thyroid gland accompanied routinely by bilateral central lymphadenectomy and, if necessary, by lateral neck lymph node dissection [1]. In the postoperative follow-up, neck ultrasonography accompanied by the calcitonin (Ct) and carcinoembrional antigen (CEA) serum level monitoring is used. Especially Ct but also CEA assessments are so sensitive, that elevated markers' serum concentration can be observed much earlier than a metastatic focus can be visualized by imaging. It has been estimated that the Ct serum level of 1000 pg/ml, which is 100 times the upper normal value limit, indicates on 1 ml of tumor tissue, although this ratio is variable [2]. Nevertheless, Ct estimation is a good measure of tumor volume.

Elevated plasma Ct levels and more rarely serum CEA concentration in some patients remain stable throughout several years, whereas in others they raise rapidly. The dynamics of these changes can be assessed by calculating the time, they need to double - their doubling time (DT). In the literature this parameter is considered as having important prognostic value in postoperative patients with MTC. One of the first publications in which the prognostic value of Ct DT was assessed was the study of Miyauchi i wsp. [3], where they found that in contrast to preoperative Ct serum level, Ct DT had an important prognostic role in overall and disease free survival prediction. Barbet et al. [4] reported that Ct and CEA DT had greater prognostic value in MTC patients than TNM or EORTC staging. Giraudet et al. [5] proved that contrary to absolute Ct and CEA concentrations it was Ct and CEA DT that was significantly correlated with the risk of progression according to the RECIST criteria. In the recently published meta-analysis, Meijer et al. [6] found Ct and CEA DT as significant risk factors for recurrence and death due to MTC. The authors used several cut-off values to classify patients as high- or low-risk and the greatest prognostic value was observed for the cut-off value of 1 year. What is interesting, they reported greater prognostic value for CEA DT compared to Ct DT.

## Patients and methods

We observed retrospectively 110 patients followed up in the Department of Nuclear Medicine and Endocrine Oncology in the Institute of Oncology, Gliwice Branch, Poland during the years 2004-2005. The Ct and CEA concentration dynamics was analyzed from the diagnosis until the most recent control visit. In 40 patients (36%) the markers never exceeded the upper limit of normal values. In the remaining 70 patients either Ct or CEA and frequently both were elevated. For these patients the DTs were calculated.

Plasma Ct concentration was assayed using the IRMA hCT kit (Cis-Bio International, Gif-sur-Yvette, France) (detection limit 1.5 pg/ml; normal range < 10 pg/ml; expected linearity up to 1500 pg/ml). Appropriate dilutions were prepared for concentrations above 200 pg/ml. CEA was measured with ARCHITECT 8200 CEA Reagent kit (Abbott, Il, USA) (sensitivity 0.5 ng/ml; normal range <5 ng/ml; expected linearity up to 1500 ng/ml).

For each patient the DTs were calculated upon the b parameter in the exponential regression equation c = a exp(b*t), where c is concentration and t is time. The DT value is equal to DT = ln 2/b, where ln 2 is the natural logarithm of 2. DT unit is the same as of time. DTs were calculated for each marker separately and the less favorable DT was specified for each patient.

Overall survival and disease-free survival was visualized on Kaplan-Meier plots and compared using Cox proportional hazard model between groups that had Ct DT, CEA DT or less-favorable DT positive and less than 2 years (group 1) versus negative or more than 2 years (group 2).

## Results

After initial total thyroidectomy (and lymph node dissection, when indicated) the concentration of both markers remained within normal range in 40 (36%) patients during follow-up. In the remaining group of 70 patients there were 46 women and 24 men. The median age at initial diagnosis was 45.5 years, it ranged from 13.4 to 74.2 years. Within that group in 24 patients sporadic MTC was diagnosed on the basis of RET diagnostics in germ-line DNA, in 16 hereditary MTC was diagnosed, whereas in 30 the genetic background is still being diagnosed or was unknown due to the lack of patient's consent. Median follow-up time was 8.25 years, ranging from 1.48 to 20.03 years. During follow-up, in 45 patients recurrence was confirmed and 23 patients died.

During the observation period significant change in DT value was noticed in no patient.

The most and the least-favorable Ct DTs was -0,65 and 0.13 years, respectively, with the 1/DT median value reflecting the DT of 4.34 years. For the CEA the respective values were -0.74, 0.11 and 4.84 years, respectively.

**Figure 1:**
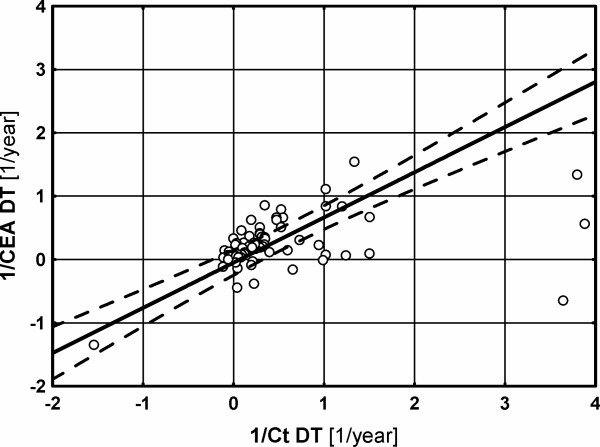
**The correlation between inverse values of Ct and CEA DT (progression rates; p < 0.0001, R2 = 0.60, slope = 0.713)**.

There was a significant correlation between the inverse values of Ct DT and CEA DT (p < 0.0001, R^2 ^= 0.60, slope = 0.713, Figure 1).

**Figure 2:**
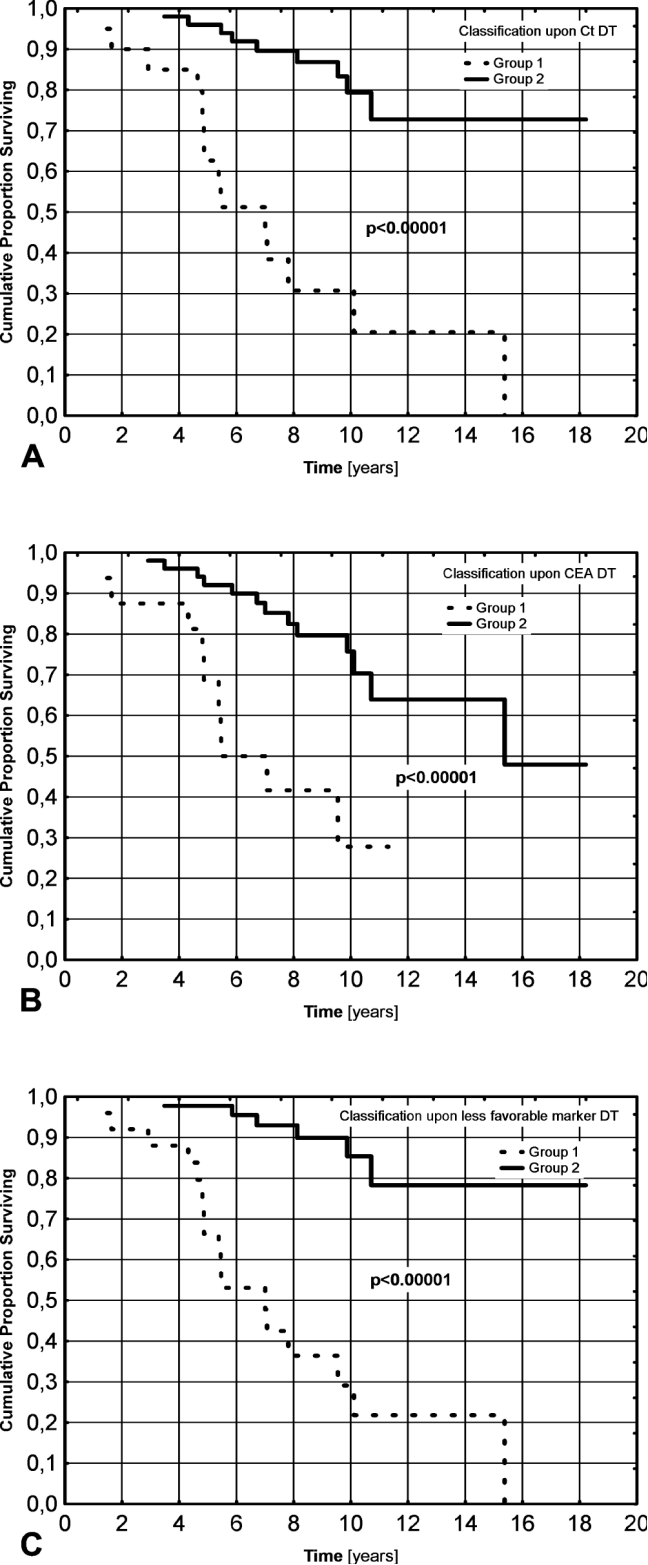
**Kaplan-Meier curves showing overall survival (A-C) between groups classified upon Ct DT (A), CEA DT (B) and less-favorable marker DT (C)**.

**Figure 3:**
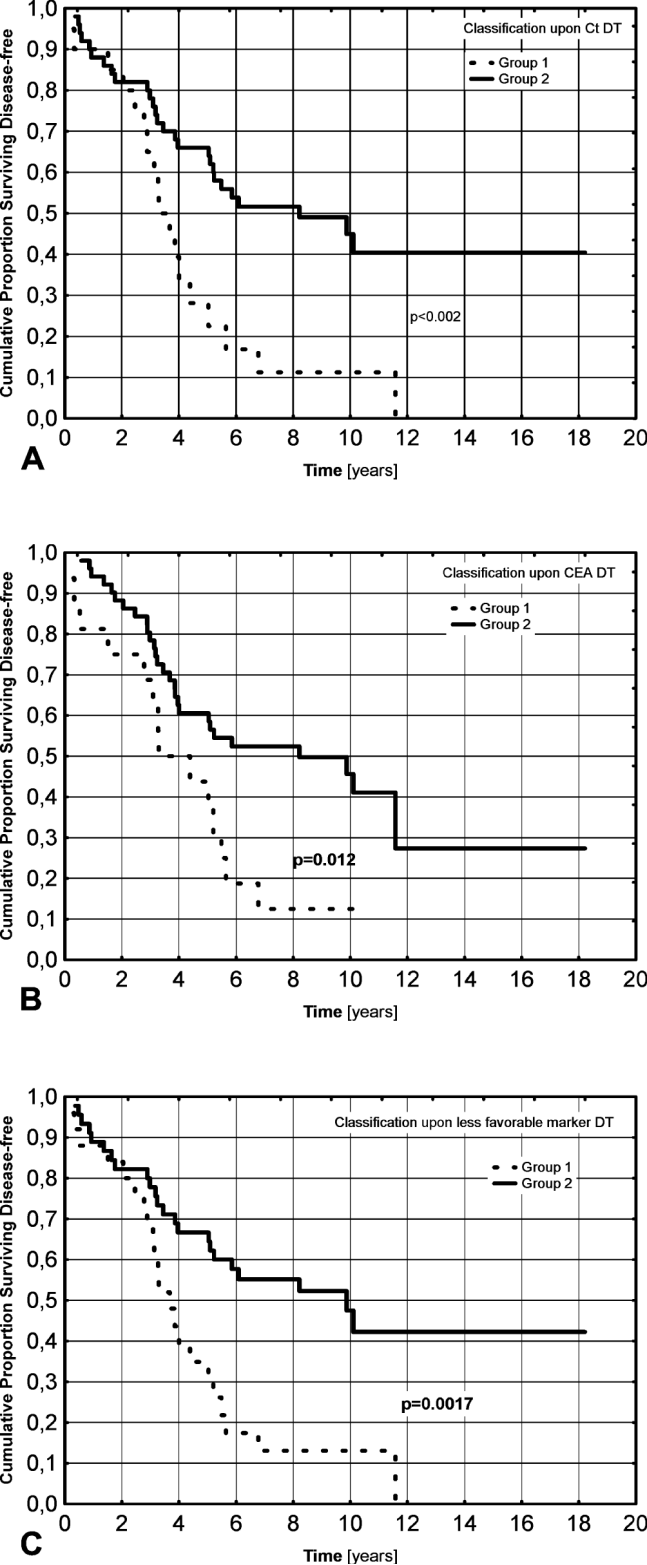
**Kaplan-Meier curves showing disease-free survival (A-C) between groups classified upon Ct DT (A), CEA DT (B) and less-favorable marker DT (C)**.

There was a significant difference between the groups distinguished upon Ct, CEA and less-favorable marker doubling times, both for overall survival, HR = 6.83 (2.92-15.97) p < 0.00001; HR = 4.22 (1.80-9.90) p = 0.0009 and HR = 8.99 (3.51-23.04) p < 0.00001, respectively (Figure 2.), and for disease-free survival HR = 2.60 (1.41-4.81) p = 0.002; HR = 2.29 (1.20-4.40) p = 0.012 and HR = 2.61 (1.43-4.71) p = 0.0017, respectively (Figure 3). The greatest hazard ratios were obtained for the classification based upon the less-favorable DT.

Group 1 in all classifications showed greater risk for death or recurrence. After 5 and 10 years of observation in groups distinguished upon less-favorable DT the rates were for group 1: 34.9 and 13.0% for recurrence-free survival and 66.3 and 29.1% for overall survival, respectively and for group 2: 66.6 and 47.5% for recurrence-free survival and 97.7 and 85.3% for overall survival, respectively. In the high-risk group, there were 4 patients with DT < 0.5 year, who all died before 5 years from diagnosis.

## Discussion

We analyzed the time-course of Ct and CEA concentrations in a group of surgically treated MTC patients followed up in a single institution. We consider the group as representative of general MTC patients population, as after the diagnosis, most of them are referred to our centre from several surgical clinics. For the majority of patients, the primary surgical treatment was outside our centre, however, after the referral the subsequent follow-up was conducted according to the strict rules resembling recently published ATA guidelines [1]. Ct and CEA estimations were performed at regular frequency, at least yearly, using the same method. Following detection of raised Ct and/or CEA level imaging was instituted, encompassing neck sonography, chest and abdominal CT and in rare occasions MR, scintigraphies and PET scans. The results obtained in our population regarding the risk of MTC recurrence are in good concordance with that published by Meijer et al. [6]. They reported recurrence HR of 2.90 (1.16-7.21) for the same classification of Ct DT as in our group. Their results concerning CEA DT hazard ratio of 4.73 (1.49-14.97) are higher than ours, but they assessed CEA DT in only 38 of their 73 patients. Their HR for total survival is also higher than in our population because they analyzed tumor-specific death, whereas such information was not available for our patients at this preliminary analysis.

CEA is generally considered to be less specific marker of MTC. The available information directly comparing Ct and CEA DT is very sparse. Barbet et al. [4] found that progression rates for Ct and CEA were well correlated, but Ct DT had greater prognostic value compared to CEA DT in multivariate analysis. On the contrary, Meijer et al. [6] in their meta-analysis found CEA DT to have more pronounced impact on the prognosis, but as previously mentioned their CEA DT results were obtained in a small group of patients. Our results suggest that Ct DT has indeed a greater prognostic value, but we found also that the classification based upon less-favorable DT had even greater prognostic meaning. This can be the result of the presence of the second, less differentiated MTC cell population, which produce preferentially CEA with short DT in addition to primary more differentiated one which is responsible for more stable Ct elevation with longer DT.

The reliable DT assessment require several marker serum level measurements spanning through sufficiently long period (optimally more than 2 years) without any therapeutic intervention. Optimally, the estimation should be performed using the same method, preferably in the same laboratory. This condition was fulfilled in our patients. In order to use it, one should not delay the serial Ct and CEA measurements after surgery. According to Brauckhoff et al. [7], reliable Ct assessments are obtained one month after thyroidectomy in almost every patient. Notably, in high-risk patients, with short DT, we can obtain reliable DT estimation much more earlier - after one or two DTs, so when the post-surgery Ct or CEA concentration is elevated, subsequent measurements should be taken in relatively short intervals, e.g. 1, then 2 and 4 months, adjusted if necessary, to reveal such high-risk individuals and consider possible treatment options. In patients with postsurgery markers' concentrations within normal range it is reasonable to take the next measurements every 3 to 6 months, depending on the stage and time elapsed from surgery. Even in the worst case scenario, when post-surgery Ct is 9.9 pg/ml and DT is 1.32 months (the lowest observed value in our group), after that time we get Ct around 233 pg/ml - the concentration at which the identification of metastatic lesion by imaging methods is still unlikely.

## Conclusions

The calcitonin and carcinembrional antigen doubling times of less than two years are negative prognostic factors for MTC recurrence-free and total survival in patients with persistent or recurrent disease. They may be used as predictive factors for more intensive search of disease localization in asymptomatic hypercalcitoninemia and for therapy choice in symptomatic disease.

## List of abbreviations

MTC: medullary thyroid carcinoma; CEA: carcinoembrional antigen; Ct: calcitonin; DT: doubling time.

## Competing interests

The authors declare that they have no competing interests.

## Authors' contributions

All authors equally contributed to the preparation of the manuscript.
